# Supplementary figures and images for: Unravelling Chlamydia trachomatis diversity in Amhara, Ethiopia: MLVA-ompA sequencing as a molecular typing tool for trachoma
Source: PLoS Negl Trop Dis. 2024 Apr 25;18(4):e0012143. doi: 10.1371/journal.pntd.0012143 (PMC11075894; doi:10.1371/journal.pntd.0012143)

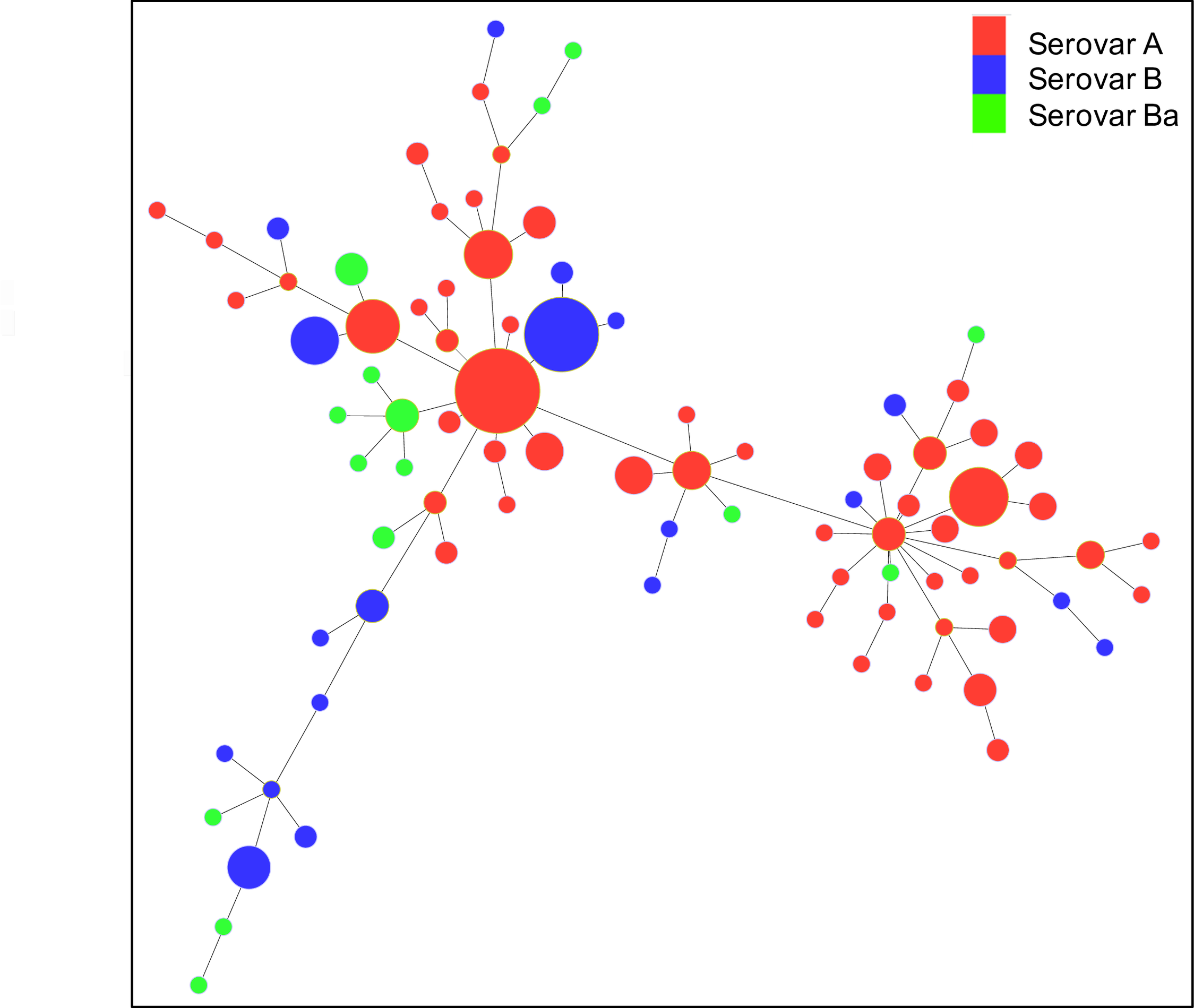

Supplement: S1 Fig — Each MLVA-ompA sequence-type (n = 87) is coloured by serovar. Each circle represents a different sequence-type, with the size of circle being directly proportional to the number of individuals who had that variant. Trees were generated using Phyloviz 2.0. (TIF) [file pntd.0012143.s001.tif]

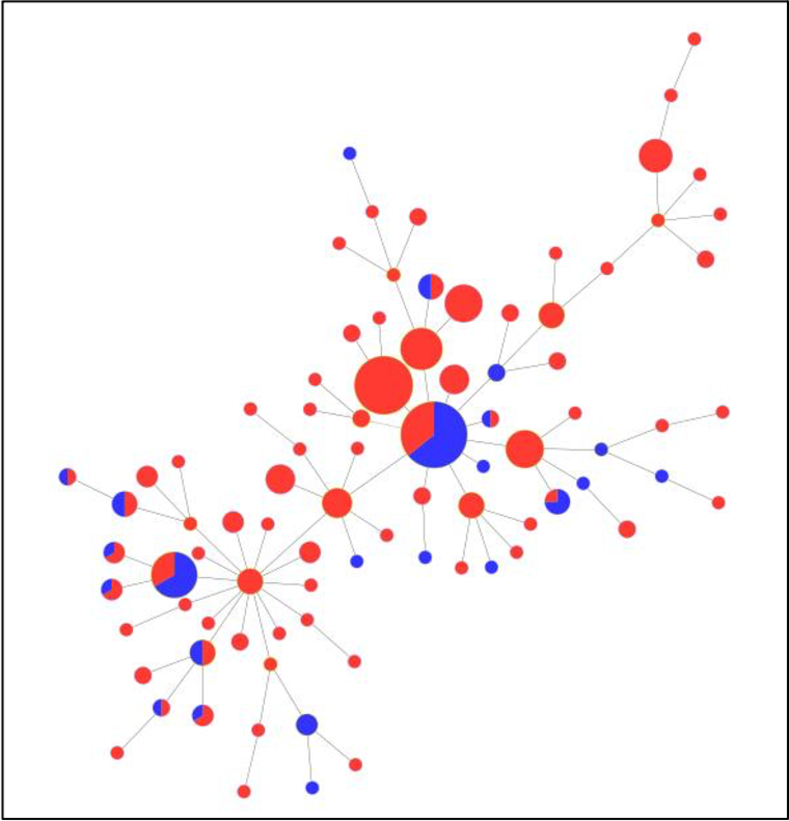

Supplement: S2 Fig — Each MLVA-ompA sequence-type (n = 87) is coloured by number of rounds of MDA, either 5 rounds (red) or 8–10 rounds (blue). Each circle represents a different sequence-type, with the size of circle being directly proportional to the number of individuals who had that variant. Trees were generated using Phyloviz 2.0. (TIF) [file pntd.0012143.s002.tif]

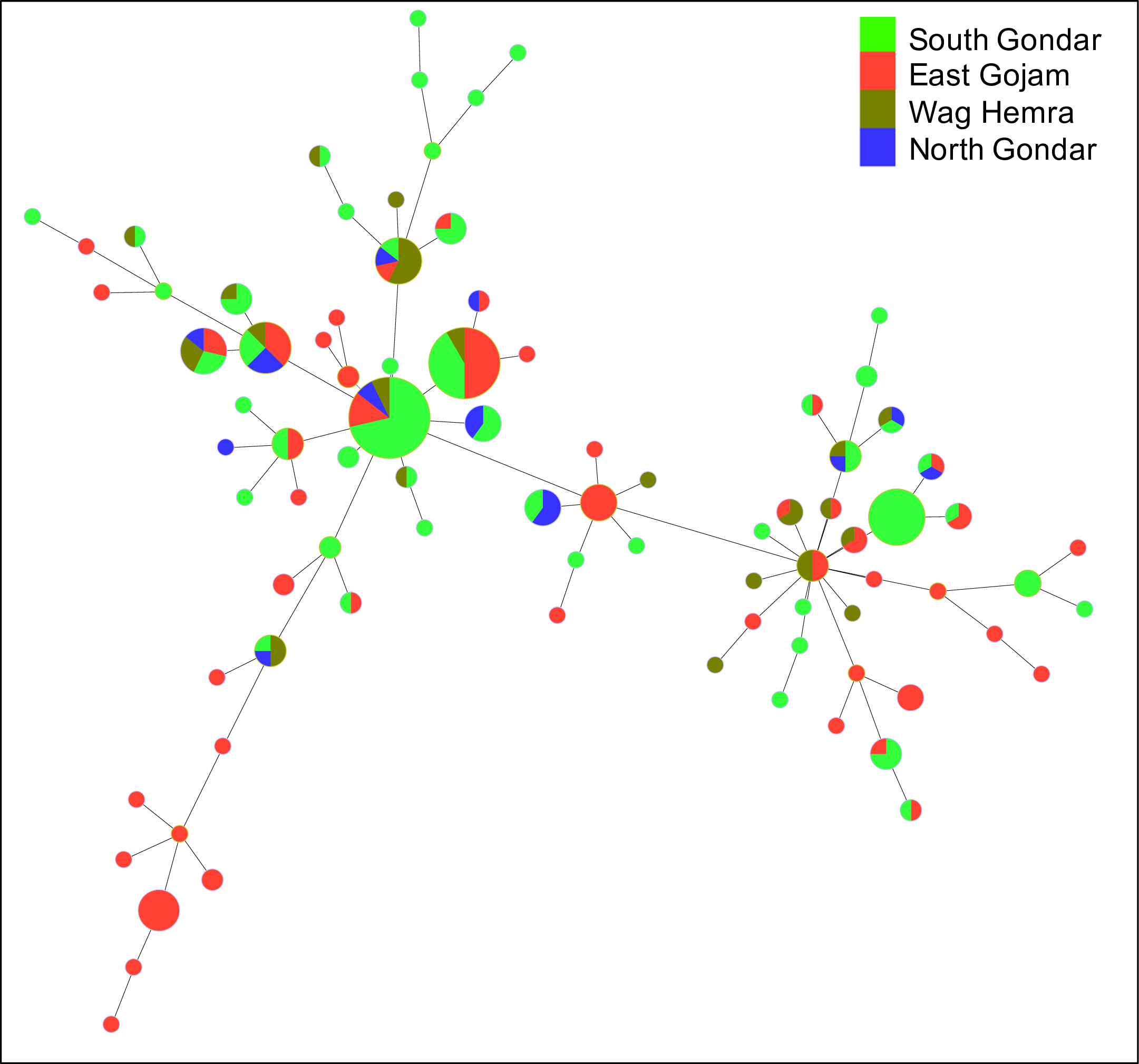

Supplement: S3 Fig — Each MLVA-ompA sequence-type (n = 87) is coloured by zone. Each circle represents a different sequence-type, with the size of circle being directly proportional to the number of individuals who had that variant. Trees were generated using Phyloviz 2.0. (TIF) [file pntd.0012143.s003.tif]
