# Supplementary material for: Unravelling Chlamydia trachomatis diversity in Amhara, Ethiopia: MLVA-ompA sequencing as a molecular typing tool for trachoma
Source: PLoS Negl Trop Dis. 2024 Apr 25;18(4):e0012143. doi: 10.1371/journal.pntd.0012143 (PMC11075894; doi:10.1371/journal.pntd.0012143)
Supplement: S1 Table — VNTR: Variable number tandem repeats. (DOCX) [file pntd.0012143.s004.docx]

**S1 table**. The total number of Ethiopian conjunctival samples unable to be sequenced using Sanger sequencing for any of the three VNTRs and/or *omp*A for any reason (denoted as an “NA”), split by year of collection. VNTR: Variable number tandem repeats.

| **Year** | **Total samples** | **Total NAs** | **Proportion** |
| --- | --- | --- | --- |
| 2004 | 69 | 30 | 0.43 |
| 2005 | 51 | 23 | 0.45 |
| 2006 | 100 | 28 | 0.28 |
| 2007 | 56 | 18 | 0.32 |
| 2008 | 14 | 4 | 0.29 |
| 2010 | 10 | 3 | 0.30 |
