# Supplementary material for: Unravelling Chlamydia trachomatis diversity in Amhara, Ethiopia: MLVA-ompA sequencing as a molecular typing tool for trachoma
Source: PLoS Negl Trop Dis. 2024 Apr 25;18(4):e0012143. doi: 10.1371/journal.pntd.0012143 (PMC11075894; doi:10.1371/journal.pntd.0012143)
Supplement: S2 Table — WGS: Whole genome sequencing. VNTR: Variable number tandem repeat. (DOCX) [file pntd.0012143.s005.docx]

**S2 table.** WGS results from 99 samples vs Sanger sequencing results for the three VNTRs CT1291, CT1299 and CT1335. WGS: Whole genome sequencing. VNTR: Variable number tandem repeat.

| **ID** | **CT1291_WGS** | **CT1291_Sanger** | **CT1335_WGS** | **CT1335_Sanger** | **CT1299_WGS** | **CT1299_Sanger** |
| --- | --- | --- | --- | --- | --- | --- |
| **1459-6700** | NA | AAATGGTCT_10C | GAAAAAGG_10T/8A | GAAAAGG_10T/8A | ATTCT_NA_ | ATTCT_10C_ |
| **1496-6885** | AAATGGTCT_9C | AAATGGTCT_NA | GAAAAAGG_10T/8A | GAAAAAGG_NA | ATTCT_14C_ | NA |
| **1524-7024** | AAATGGTCT_9C | AAATGGTCT_10C | GAAAAAGG_10T/8A | GAAAAGG_10T/8A | ATTCT_14C_ | ATTCT_10C_ |
| **1612-7464** | AAATGGTCT_10C | AAATGGTCT_9C | GAAAAAGG_10T/8A | GAAAAGG_10T/8A | NA | ATTCT_14C_ |
| **1623-7519** | AAATGGTCT_10C | AAATGGTCT_10C | GAAAAAGG_10T/8A | GAAAAAGG_10T/8A | NA | ATTCT_13C_ |
| **1629-7546** | AAATGGTCT_10C | AAATGGTCT_9C | GAAAAAGG_10T/8A | GAAAAGG_10T/8A | NA | ATTCT_10C_ |
| **1630-7550** | AAATGGTCT_9C | AAATGGTCT_9C | GAAAAAGG_10T/8A | GAAAAGG_10T/8A | ATTCT_14C_ | ATTCT_11C_ |
| **1633-7568** | AAATGGTCT_9C | AAATGGTCT_9C | GAAAAAGG_10T/8A | GAAAAGG_10T/8A | NA | ATTCT_11C_ |
| **1653-7666** | AAATGGTCT_10C | AAATGGTCT_NA | GAAAAAGG_10T/8A | GAAAAAGG_10T/8A | NA | ATTCT_11C |
| **1658-7694** | AAATGGTCT_9C | AAATGGTCT_8C | GAAAAAGG_10T/8A | GAAAAGG_10T/8A | NA | ATTCT_14C_ |
| **1667-7738** | AAATGGTCT_10C | AAATGGTCT_9C | GAAAAAGG_10T/8A | GAAAAGG_10T/8A | ATTCT_14C_ | ATTCT_10C_ |
| **1675-7776** | AAATGGTCT_9C | AAATGGTCT_9C | GAAAAAGG_10T/8A | GAAAAGG_10T/8A | ATTCT_14C_ | ATTCT_12C_ |
| **1680-7799** | AAATGGTCT_9C | AAATGGTCT_9C | GAAAAAGG_10T/8A | GAAAAGG_10T/8A | ATTCT_14C_ | ATTCT_10C_ |
| **1682-7810** | AAATGGTCT_10C | AAATGGTCT_9C | GAAAAAGG_10T/8A | GAAAAAGG_10T/8A | NA | ATTCT_11C_ |
| **1689-7847** | AAATGGTCT_10C | AAATGGTCT_9C | GAAAAAGG_10T/8A | GAAAAAGG_10T/8A | ATTCT_14C_ | ATTCT_10C_ |
| **1710-7953** | AAATGGTCT_9C | AAATGGTCT_9C | GAAAAAGG_10T/8A | GAAAAAGG_NA | NA | ATTCT_10C |
| **1767-8232** | AAATGGTCT_9C | AAATGGTCT_10C | GAAAAAGG_10T/8A | GAAAAGG_10T/8A | NA | ATTCT_14C_ |
| **1770-8246** | NA | AAATGGTCT_NA | GAAAAAGG_10T/8A | GAAAAAGG_10T/8A | NA | ATTCT_14C |
| **1773-8261** | AAATGGTCT_9C | AAATGGTCT_9C | GAAAAAGG_10T/8A | GAAAAGG_10T/8A | ATTCT_14C_ | ATTCT_11C_ |
| **1781-8301** | AAATGGTCT_9C | AAATGGTCT_9C | GAAAAAGG_10T/8A | GAAAAAGG_10T/8A | ATTCT_14C_ | ATTCT_15C |
| **1801-8400** | AAATGGTCT_9C | AAATGGTCT_10C | GAAAAAGG_10T/8A | GAAAAGG_10T/8A | ATTCT_14C_ | ATTCT_11C_ |
| **1859-8691** | AAATGGTCT_10C | AAATGGTCT_9C | GAAAAAGG_10T/8A | GAAAAGG_10T/8A | ATTCT_14C_ | ATTCT_10C_ |
| **1866-8726** | AAATGGTCT_9C | AAATGGTCT_8C | GAAAAAGG_10T/8A | GAAAAAGG_10T/8A | ATTCT_14C_ | NA |
| **1870-8742** | AAATGGTCT_9C | AAATGGTCT_9C | GAAAAAGG_10T/8A | GAAAAAGG_10T/8A | NA | ATTCT_15C |
| **1878-8783** | AAATGGTCT_NA | AAATGGTCT_9C | GAAAAAGG_10T/8A | GAAAAGG_10T/8A | NA | ATTCT_10C_ |
| **1879-8790** | AAATGGTCT_10C | AAATGGTCT_9C | GAAAAAGG_10T/8A | GAAAAGG_10T/8A | ATTCT_14C_ | ATTCT_10C_ |
| **2134-9948** | AAATGGT_12C | AAATGGT_8C | GAAAAAGG_10T/8A | GAAAAGG_10T/8A | ATTCTATTCT_9C_ | ATTCT_9C_ |
| **2136-9960** | AAATGGTCT_9C | AAATGGTCT_9C | GAAAAAGG_10T/8A | GAAAAGG_10T/8A | NA | ATTCT_11C_ |
| **2137-9962** | NA | AAATGGT_8C | GAAAAAGG_10T/8A | GAAAAAGG_10T/8A | ATTCT_14C_ | ATTCT_9C |
| **2148-10019** | AAATGGTCT_9C | AAATGGTCT_9C | GAAAAAGG_10T/8A | GAAAAGG_10T/8A | ATTCT_14C_ | ATTCT_10C_ |
| **2149-10022** | AAATGGTCT_9C | AAATGGTCT_8C | GAAAAAGG_10T/8A | GAAAAGG_10T/8A | ATTCT_14C_ | ATTCT_10C_ |
| **2166-10103** | AAATGGTCT_9C | AAATGGTCT_8C | GAAAAAGG_10T/8A | GAAAAGG_10T/8A | ATTCT_14C_ | ATTCT_16C |
| **2166-10106** | AAATGGTCT_9C | AAATGGTCT_9C | GAAAAAGG_10T/8A | GAAAAGG_10T/8A | ATTCT_14C_ | ATTCT_11C_ |
| **2169-10121** | AAATGGTCT_9C | AAATGGTCT_8C | GAAAAAGG_10T/8A | GAAAAGG_10T/8A | ATTCT_14C_ | ATTCT_10C_ |
| **2174-10147** | AAATGGTCT_9C | AAATGGTCT_8C | GAAAAAGG_10T/8A | GAAAAAGG_10T/8A | NA | ATTCT_14C |
| **2175-10152** | AAATGGTCT_9C | AAATGGTCT_8C | GAAAAAGG_10T/8A | GAAAAGG_10T/8A | ATTCT_3CT_10C_ | ATTCT_10C_ |
| **2178-10163** | AAATGGTCT_9C | AAATGGTCT_9C | GAAAAAGG_NT/8A | GAAAAGG_9T/8A | ATTCT_14C_ | ATTCT_9C_ |
| **2182-10186** | AAATGGTCT_9C | AAATGGTCT_8C | GAAAAAGG_10T/8A | GAAAAGG_10T/8A | ATTCT_14C_ | ATTCT_14C_ |
| **2188-10216** | AAATGGTCT_NA | AAATGGTCT_8C | GAAAAAGG_10T/8A | GAAAAGG_10T/8A | ATTCT_14C_ | ATTCT_13C_ |
| **2209-10263** | AAATGGTCT_NA | AAATGGTCT_8C | GAAAAAGG_NT/8A | GAAAAGG_9T/8A | ATTCT_14C_ | ATTCT_11C_ |
| **2228-10360** | AAATGGTCT_NA | AAATGGTCT_8C | GAAAAAGG_10T/8A | GAAAAGG_10T/8A | ATTCT_14C_ | ATTCT_10C_ |
| **2233-10384** | AAATGGTCT_NA | AAATGGTCT_8C | GAAAAAGG_10T/8A | GAAAAGG_10T/8A | ATTCT_14C_ | ATTCT_10C_ |
| **2241-10425** | AAATGGT_12C | AAATGGT_8C | GAAAAAGG_10T/8A | GAAAAAGG_NA | NA | ATTCT_9C |
| **2243-10433** | AAATGGT_NA | AAATGGTCT_8C | GAAAAAGG_10T/8A | GAAAAAGG_10T/8A | ATTCTCTTCT_9C_ | ATTCT_10C_ |
| **2243-10434** | AAATGGT_12C | AAATGGT_8C | GAAAAAGG_10T/8A | GAAAAGG_10T/8A | ATTCTATTCT_9C_ | ATTCT_9C_ |
| **2247-10454** | AAATGGT_NA | AAATGGT_8C | GAAAAAGG_10T/8A | GAAAAGG_10T/8A | ATTCT_14C_ | ATTCT_10C_ |
| **2247-10456** | AAATGGTCT_10C | AAATGGTCT_9C | GAAAAAGG_10T/8A | GAAAAGG_10T/8A | ATTCT_14C_ | ATTCT_10C_ |
| **2248-10461** | NA | AAATGGTCT_9C | GAAAAAGG_10T/8A | GAAAAGG_10T/8A | NA | ATTCT_10C_ |
| **2251-10472** | AAATGGT_NA | AAATGGT_9C | GAAAAAGG_10T/8A | GAAAAAGG_10T/8A | NA | ATTCT_10C_ |
| **2254-10487** | AAATGGT_12C | AAATGGT_8C | GAAAAAGG_10T/8A | GAAAAGG_10T/8A | ATTCT_14C_ | ATTCT_9C_ |
| **2255-10495** | AAATGGT_NA | AAATGGT_8C | GAAAAAGG_10T/8A | GAAAAGG_10T/8A | ATTCTATTCT_9C_ | ATTCT_9C_ |
| **2256-10501** | AAATGGT_12C | AAATGGT_8C | GAAAAAGG_10T/8A | GAAAAGG_10T/8A | NA | ATTCT_9C_ |
| **2258-10504** | AAATGGTCT_9C | AAATGGTCT_9C | GAAAAAGG_10T/8A | GAAAAGG_10T/8A | NA | ATTCT_10C_ |
| **2268-10556** | AAATGGTCT_9C | AAATGGTCT_9C | GAAAAAGG_NT/8A | GAAAAGG_9T/8A | NA | ATTCT_9C_ |
| **2280-10615** | AAATGGTCT_10C | AAATGGTCT_9C | GAAAAAGG_10T/8A | GAAAAGG_10T/8A | NA | ATTCT_11C_ |
| **2294-10681** | AAATGGTCT_9C | AAATGGTCT_7C | GAAAAAGG_10T/8A | GAAAAAGG_10T/8A | ATTCT_14C_ | ATTCT_11C_ |
| **2303-10728** | AAATGGTCT_9C | AAATGGTCT_8C | GAAAAAGG_10T/8A | GAAAAGG_10T/8A | ATTCT_14C_ | ATTCT_12C_ |
| **2314-10783** | AAATGGTCT_NA | AAATGGTCT_9C | GAAAAAGG_10T/8A | GAAAAGG_10T/8A | ATTCT_14C_ | ATTCT_10C_ |
| **2356-10989** | AAATGGTCT_9C | AAATGGTCT_9C | GAAAAAGG_10T/8A | GAAAAGG_10T/8A | ATTCT_14C_ | ATTCT_10C_ |
| **2394-11155** | AAATGGTCT_9C | AAATGGTCT_9C | GAAAAAGG_10T/8A | GAAAAGG_10T/8A | NA | ATTCT_10C_ |
| **2405-11212** | NA | AAATGGTCT_11C | GAAAAAGG_10T/8A | GAAAAGG_10T/8A | NA | ATTCT_14C_ |
| **2406-11215** | NA | AAATGGTCT_NA | GAAAAAGG_10T/8A | GAAAAGG_10T/8A | NA | ATTCT_14C |
| **2437-11366** | NA | AAATGGTCT_11C | GAAAAAGG_10T/8A | GAAAAGG_10T/8A | ATTCT_14C_ | ATTCT_14C_ |
| **2441-11387** | AAATGGTCT_9C | AAATGGTCT_9C | GAAAAAGG_10T/8A | GAAAAGG_10T/8A | NA | ATTCT_11C_ |
| **2451-11436** | AAATGGTCT_10C | AAATGGTCT_9C | GAAAAAGG_10T/8A | GAAAAGG_10T/8A | ATTCT_14C_ | ATTCT_10C_ |
| **2452-11442** | AAATGGTCT_10C | AAATGGTCT_9C | GAAAAAGG_10T/8A | GAAAAAGG_10T/8A | ATTCT_14C_ | ATTCT_9C_ |
| **2495-11650** | AAATGGTCT_9C | AAATGGT_9C | GAAAAAGG_10T/8A | GAAAAGG_10T/8A | ATTCT_14C_ | ATTCT_10C_ |
| **2495-11652** | AAATGGT_12C | AAATGGTCT_8C | GAAAAAGG_10T/8A | GAAAAAGG_NA | ATTCT_14C_ | ATTCT_9C |
| **2496-11655** | AAATGGTCT_10C | AAATGGTCT_9C | GAAAAAGG_10T/8A | GAAAAAGG_NA | ATTCT_14C_ | ATTCT_10C |
| **2972-13924** | AAATGGTCT_10C | AAATGGTCT_NA | GAAAAAGG_10T/8A | GAAAAAGG_NA | ATTCT_14C_ | NA |
| **2976-13947** | AAATGGTCT_9C | AAATGGTCT_9C | GAAAAAGG_10T/8A | GAAAAGG_10T/8A | ATTCT_14C_ | ATTCT_11C_ |
| **2983-13981** | AAATGGTCT_10C | AAATGGTCT_9C | GAAAAAGG_10T/8A | GAAAAAGG_10T/8A | ATTCT_14C_ | ATTCT_10C_ |
| **2987-13999** | AAATGGTCT_9C | AAATGGTCT_9C | GAAAAAGG_10T/8A | GAAAAGG_10T/8A | ATTCT_14C_ | ATTCT_11C_ |
| **2991-14016** | AAATGGTCT_10C | AAATGGTCT_9C | GAAAAAGG_10T/8A | GAAAAGG_10T/8A | ATTCT_14C_ | ATTCT_11C_ |
| **2994-14032** | AAATGGTCT_9C | AAATGGTCT_9C | GAAAAAGG_10T/8A | GAAAAGG_10T/8A | ATTCT_14C_ | ATTCT_10C_ |
| **3002-14069** | NA | AAATGGTCT_10C | GAAAAAGG_10T/8A | GAAAAGG_10T/8A | ATTCT_14C_ | ATTCT_10C_ |
| **3004-14083** | NA | AAATGGT_8C | GAAAAAGG_10T/8A | GAAAAGG_10T/8A | ATTCTCTTCT_9C_ | ATTCT_10C_ |
| **3009-14104** | AAATGGTCT_9C | AAATGGTCT_9C | GAAAAAGG_10T/8A | GAAAAAGG_10T/8A | NA | ATTCT_15C |
| **3027-14195** | AAATGGTCT_9C | AAATGGTCT_9C | GAAAAAGG_10T/8A | GAAAAGG_10T/8A | NA | ATTCT_13C_ |
| **3030-14213** | AAATGGTCT_9C | AAATGGTCT_9C | GAAAAAGG_10T/8A | GAAAAGG_10T/8A | NA | ATTCT_15C |
| **3037-14244** | AAATGGTCT_9C | AAATGGTCT_9C | GAAAAAGG_10T/8A | GAAAAAGG_NA | NA | ATTCT_14C |
| **32-154** | AAATGGTCT_10C | AAATGGTCT_9C | GAAAAAGG_10T/8A | GAAAAAGG_10T/8A | ATTCT_14C_ | ATTCT_10C_ |
| **42-207** | AAATGGTCT_10C | AAATGGTCT_9C | GAAAAAGG_10T/8A | GAAAAGG_10T/8A | ATTCT_14C_ | ATTCT_10C_ |
| **45-216** | AAATGGTCT_9C | AAATGGTCT_9C | GAAAAAGG_10T/8A | GAAAAGG_10T/8A | ATTCT_14C_ | ATTCT_13C_ |
| **45-218** | AAATGGTCT_9C | AAATGGTCT_9C | GAAAAAGG_10T/8A | GAAAAGG_10T/8A | ATTCT_14C_ | ATTCT_11C_ |
| **62-303** | AAATGGTCT_9C | AAATGGTCT_8C | GAAAAAGG_10T/8A | GAAAAAGG_10T/8A | NA | ATTCT_18C |
| **64-311** | AAATGGTCT_9C | AAATGGTCT_8C | GAAAAAGG_10T/8A | GAAAAGG_10T/8A | NA | ATTCT_14C_ |
| **65-315** | AAATGGTCT_9C | AAATGGTCT_8C | GAAAAAGG_10T/8A | GAAAAAGG_10T/8A | NA | ATTCT_16C |
| **71-349** | AAATGGTCT_9C | AAATGGTCT_12C | GAAAAAGG_10T/8A | GAAAAAGG_NA | ATTCT_14C_ | ATTCT_14C |
| **75-367** | AAATGGTCT_9C | AAATGGTCT_12C | GAAAAAGG_10T/8A | GAAAAGG_10T/8A | ATTCT_14C_ | ATTCT_10C_ |
| **850-3968** | AAATGGTCT_9C | AAATGGTCT_9C | GAAAAAGG_10T/8A | GAAAAGG_10T/8A | ATTCT_14C_ | ATTCT_10C_ |
| **865-4011** | AAATGGTCT_9C | AAATGGTCT_9C | GAAAAAGG_10T/8A | GAAAAGG_10T/8A | ATTCT_14C_ | ATTCT_11C_ |
| **865-4012** | AAATGGTCT_10C | AAATGGTCT_9C | GAAAAAGG_10T/8A | GAAAAAGG_NA | NA | ATTCT_11C |
| **866-4014** | AAATGGTCT_10C | AAATGGTCT_9C | GAAAAAGG_10T/8A | GAAAAAGG_10T/8A | ATTCT_14C_ | ATTCT_10C_ |
| **872-4044** | AAATGGTCT_10C | AAATGGTCT_9C | GAAAAAGG_10T/8A | GAAAAGG_10T/8A | NA | ATTCT_11C_ |
| **873-4051** | AAATGGTCT_10C | AAATGGTCT_9C | GAAAAAGG_10T/8A | GAAAAAGG_NA | ATTCT_14C_ | ATTCT_11C |
| **884-4103** | AAATGGTCT_10C | AAATGGTCT_9C | GAAAAAGG_10T/8A | GAAAAAGG_10T/8A | ATTCT_14C_ | ATTCT_11C_ |
| **889-4129** | AAATGGTCT_9C | AAATGGTCT_9C | GAAAAAGG_10T/8A | GAAAAGG_10T/8A | ATTCT_14C_ | ATTCT_13C_ |
| **889-4130** | AAATGGTCT_9C | AAATGGTCT_9C | GAAAAAGG_10T/8A | GAAAAGG_10T/8A | ATTCT_14C_ | ATTCT_13C_ |
