# Supplementary material for: Unravelling Chlamydia trachomatis diversity in Amhara, Ethiopia: MLVA-ompA sequencing as a molecular typing tool for trachoma
Source: PLoS Negl Trop Dis. 2024 Apr 25;18(4):e0012143. doi: 10.1371/journal.pntd.0012143 (PMC11075894; doi:10.1371/journal.pntd.0012143)
Supplement: S3 Table — (DOCX) [file pntd.0012143.s006.docx]

**S3 table**. The serovar and Pedersen sequence-type associated with each sample ID used to generate the phylogenetic trees (n=45).

| **ID** | **Pedersen Code** | **Serovar** |
| --- | --- | --- |
| 1524-7024 | 3b.4.4 | A |
| 1675-7776 | 3b.6.3 | A |
| 1680-7799 | 3b.4.3 | A |
| 1773-8261 | 3b.5.3 | A |
| 1801-8400 | 3b.5.4 | A |
| 2148-10019 | 3b.4.3 | A |
| 2149-10022 | 3b.4.2 | A |
| 2166-10106 | 3b.5.3 | A |
| 2169-10121 | 3b.4.2 | A |
| 2175-10152 | 3b.4.2 | A |
| 2178-10163 | 1a.3.3 | A |
| 2182-10186 | 3b.8.2 | A |
| 2294-10681 | 3.5.8 | A |
| 2303-10728 | 3b.6.2 | A |
| 2976-13947 | 3b.5.3 | A |
| 2987-13999 | 3b.5.3 | A |
| 2994-14032 | 3b.4.3 | A |
| 45-216 | 3b.7.3 | A |
| 45-218 | 3b.5.3 | A |
| 71-347 | 3.8.2 | A |
| 75-367 | 3b.4.7 | A |
| 850-3968 | 3b.4.3 | A |
| 889-4129 | 3b.7.3 | A |
| 889-4130 | 3b.7.3 | A |
| 1630-7550 | 3b.5.3 | B |
| 1667-7738 | 3b.4.3 | B |
| 1689-7847 | 3.4.3 | B |
| 1859-8691 | 3b.4.3 | B |
| 1879-8790 | 3b.4.3 | B |
| 2134-9948 | 3b.3.2c | B |
| 2243-10434 | 3b.3.2c | B |
| 2254-10487 | 3b.3.2c | B |
| 2356-10989 | 3b.4.3 | B |
| 2451-11436 | 3b.4.3 | B |
| 2452-11442 | 3.3.3 | B |
| 2495-11650 | 3b.4.3c | B |
| 2983-13981 | 3.4.3 | B |
| 2991-14016 | 3b.5.3 | B |
| 32-154 | 3.4.3 | B |
| 42-207 | 3b.4.3 | B |
| 865-4011 | 3b.5.3 | B |
| 866-4014 | 3.4.3 | B |
| 884-4103 | 3.5.3 | B |
