# Supplementary material for: Unravelling Chlamydia trachomatis diversity in Amhara, Ethiopia: MLVA-ompA sequencing as a molecular typing tool for trachoma
Source: PLoS Negl Trop Dis. 2024 Apr 25;18(4):e0012143. doi: 10.1371/journal.pntd.0012143 (PMC11075894; doi:10.1371/journal.pntd.0012143)
Supplement: Attachment — Submitted filename: Reviewer comments _MLVA_3.docx [file pntd.0012143.s008.docx]

22-March-2024 

Dear Dr. Ngondi and Dr Nascimento,

Thank you for the helpful comments and suggestions on our manuscript.

Please find the response to the reviewers' questions and comments below. We have updated the manuscript in response to most comments, however some of the suggestions we have not included as we did not feel the extra information requested would benefit this particular paper.

We would like to thank all three reviewers for their excellent suggestions and support, and for taking the time to help us improve this manuscript. 
 
Yours sincerely, 
 

Anna Harte

Reviewer's Responses to Questions

Key Review Criteria Required for Acceptance?
As you describe the new analyses required for acceptance, please consider the following:

Methods
-Are the objectives of the study clearly articulated with a clear testable hypothesis stated?
-Is the study design appropriate to address the stated objectives?
-Is the population clearly described and appropriate for the hypothesis being tested?
-Is the sample size sufficient to ensure adequate power to address the hypothesis being tested?
-Were correct statistical analysis used to support conclusions?
-Are there concerns about ethical or regulatory requirements being met?

Reviewer #1: The methods are excellent, except I believe that if they are trying to understand why the rates of trachoma had not fallen they should have accessed changes in facial cleanliness rates to have a measure of transmission potential.

Thank you for your review. The main focus of this study was on the effect of MDA and other interventions (such as F and E interventions as part of SAFE) on genetic diversity over time in Amhara. We have added more detail on the F and E interventions that the program delivered during this time to try to increase the prevalence of WASH indicators including facial cleanliness throughout the region. We have added this information to the methods and discussion (lines 135-137,143-144,612-615). In the Discussion we further note that while F and E indicators have increased over time, further effort is certainly needed (lines 676-678). As part of that we have now included citations where more information on WASH indicators can be found for the region (lines 617).

Reviewer #2: In the manuscript, the authors investigated the genetic diversity of Chlamydia trachomatis in Amhara, Ethiopia, and compared this diversity at two timepoints: during 5 rounds and 8-10 rounds of mass drug administration (MDA) using VNTR combined with ompA genotyping. The methodology employed in the study appears to be appropriate for the research objectives and the authors utilized sufficient statistical methods required for analyzing genetic data.

Thank you for the comments.

Reviewer #3: All appropriate. Stats could be expanded as mentioned below

Thank you for the comments.
--------------------

Results
-Does the analysis presented match the analysis plan?
-Are the results clearly and completely presented?
-Are the figures (Tables, Images) of sufficient quality for clarity?

Reviewer #1: The results are excellent

Thank you for the comments.

Reviewer #2: The analysis presented in the study aligns with the outlined analysis plan and the results are clearly and thoroughly presented. However, I have a few suggestions to further enhance the clarity and impact of the results:

1- For comparison with MLVA-ompA, it would be beneficial to include a minimum spanning tree (MST) of ompA and VNTR sequences overlaid with serovar, zone, and date of sample collection in the supplementary data.

Only ompA serotype information was used in this analysis and not variant alleles. There are numerous publications on ompA allele variation in the literature from many studies of both ocular and genital infection so the authors feel including ompA sequencing information would add little and distract from the main message of the paper which is that VNTR/MVLA offers sufficient genotyping resolution for molecular epidemiological studies in trachoma.

2- I recommend the authors provide a Tanglegram comparing tree topology differences between whole-genome sequencing (WGS) extracted and Sanger sequencing VNTR, instead of presenting two phylogeny trees in figures 5 and 6.

This has been added and figures 5 and 6 have been removed (line 478). The methods and results have been updated as appropriate.

3- It would enhance the analysis if the authors included a Tanglegram comparing MLVA-ompA sequencing versus ompA sequencing.

OmpA variant information was not used in this study and so the authors feel including ompA sequencing information would not be relevant. Many others have demonstrated that the resolution of ompA sequence alone is insufficient, such as Andreasen et al (2008) and Justrand et al (2010).

Andreasen, A.A., Burton, M.J., Holland, M.J., Polley, S., Faal, N., Mabey, D.C. and Bailey, R.L., 2008. Chlamydia trachomatis ompA variants in trachoma: what do they tell us?. PLoS neglected tropical diseases, 2(9), p.e306.

Jurstrand, M., Christerson, L., Klint, M., Fredlund, H., Unemo, M. and Herrmann, B., 2010. Characterisation of Chlamydia trachomatis by ompA sequencing and multilocus sequence typing in a Swedish county before and after identification of the new variant. Sexually transmitted infections, 86(1), pp.56-60.

Reviewer #3: Yes. could be trimmed, but not necessary for online journal?

We have kept the results section as it is in order to comprehensively explain each section.


Conclusions
-Are the conclusions supported by the data presented?
-Are the limitations of analysis clearly described?
-Do the authors discuss how these data can be helpful to advance our understanding of the topic under study?
-Is public health relevance addressed?

Reviewer #1: The conclusion that their sequencing is much cheaper that WGS is sound.
However, in the abstract and discussion there are several areas where the influence of the lack of facial cleanliness on the potential for transmission has been omitted. Lines 16-17, 42-43 and in part 61-62 gloss over the importance of facial cleanliness as the long-term measure to reduce trachoma transmission and reinfection. If in lines 573-575 they wanted to find out why rates of trachoma had not fallen or had recrudesced they should be measuring facial cleanliness.
What is totally unclear is how their sequencing will be ‘aiding in the development of targeted interventions for trachoma control’ (Lines 37,53, and much of the discussion). This is especially so as they did not find any association between STs and trachoma prevalence of intensity, Line 518-519 and in the results.

We have added more information on F (facial cleanliness) and E (environmental improvement) at the locations suggested (lines 18, 48). We have further now noted that parts of Amhara are still experiencing high TF and CT, and that WASH interventions including those that lead to facial cleanliness need to be strengthened (line 615).

We have also clarified how using this sequencing technique to improve our understanding of transmission dynamics could be useful in the development of trachoma interventions in the discussion section (lines 601, 637, 672-678).

Reviewer #2: The conclusions drawn in the study are well-supported by the data presented. The authors effectively outline the limitations of their analysis. Furthermore, the study addresses the public health relevance of its findings.

Thank you for the comments.

Reviewer #3: Could add in some limitations. a few listed below. could add in interpretation of decreased diversity. again, as below

Thank you for the suggestions, we have added the points you have outlined below.
--------------------

Editorial and Data Presentation Modifications?
Use this section for editorial suggestions as well as relatively minor modifications of existing data that would enhance clarity. If the only modifications needed are minor and/or editorial, you may wish to recommend “Minor Revision” or “Accept”.

Reviewer #1: I don’t know if the authors have facial cleanliness data but if they do they could improve this paper significantly.

As previously mentioned, the focus of this paper was on the observed genomic diversity within a region with an active trachoma control program which includes MDA and F and E interventions. Citations on those programmatic efforts have been included in the relevant locations in the manuscript (line 617). The examination of associations between specific WASH indicators and genomic diversity was beyond the scope of this manuscript. Given the limited sample size, the limited geographic range, and the relative novelty of this typing method for ocular Ct, we chose to focus on how genomic diversity was related to trachoma outcomes themselves.

Reviewer #2: 1- Please review Table 4A for any typos. I am uncertain if the last ST should be listed as 4* or 14*.

14* is correct as Pedersen et al (2008) and Wang et al (2011) identified 13 CT1335 types in total, so the novel CT1335 sequence type we have found is number 14. We have updated the table caption to highlight that only sequences found in this study are shown (line 438).

2- I couldn't find the sequences in accession number provided. Please check the accession number for typo error.

We have double checked all the accession numbers and they are correct. B/tunis864 is deposited and can be found under the ENA accession number ERR12253485 rather than NCBI which the reviewer is perhaps more familiar with?

Reviewer #3: (No Response)

--------------------

Summary and General Comments
Use this section to provide overall comments, discuss strengths/weaknesses of the study, novelty, significance, general execution and scholarship. You may also include additional comments for the author, including concerns about dual publication, research ethics, or publication ethics. If requesting major revision, please articulate the new experiments that are needed.

Reviewer #1: (No Response)

Reviewer #2: (No Response)

Reviewer #3: This thorough analysis of chlamydia variants in a trachoma-endemic area covers a number of issues: phylogenetics, minimum spanning trees, spatiotemporal clustering, etc. While these are well worth reporting, a particular point of interest is the apparent decrease in diversity with MDA, as it may have clinical ramifications. If diversity helps evade the human host response, then lower diversity post MDA may prevent chlamydia from returning to pre-treatment levels. I believe that your finding of decreased diversity is important. The authors could consider expanding on their important finding of a decrease. Either in a few further analyses, or in the discussion. Or both! Either would be of interest though.

Thank you for this comment, we have expanded on the importance of diversity in the discussion section as suggested (lines 612-638).

1) Community-level diversity across different bacterial genera and species has decreased in RCTs of azithromycin MDA. Earlier studies suggested that diversity within trachoma variants of chlamydia might be lower with lower prevalence across regions, and hypothesized that diversity might be lower post-MDA. But this was not found in at least one trachoma RCT (Chin et al, AJE, 2018). This highlights the importance of your results.

Thank you for your comment, this is an important point and we have added text to the discussion to highlight and compare the findings of this study with those in the literature such as Chin et al. 2018 (line 629).

2) The high discriminatory power (which I think is synonymous with Simpson’s diversity or 1-Simpson’s index if I understand correctly) and your Figure 7 imply the vast majority of variants were unique. The most information about diversity can sometimes be obtained by operational taxonomic units that are neither too broad (can’t distinguish differences) nor too narrow (find every sample is unique, as found in Guinea Bissau). You have the opportunity to try several operational taxonomic units of different discriminatory power. I may have missed, but did you compare pre- and post-MDA with each of your diversity measures?

The only diversity measure we used was Simpson’s diversity, which was compared between pre-and post MDA timepoints using Hutcheson’s T-test for the zone that had repeated sampling (figure 6).

3) Fine to display as 1-Simpson’s index, with interpretation as your discriminatory power (again, if I understand correctly). Another natural unit is as “effective number”, which would be the reciprocal of Simpson’s index (L-2 Renyi entropy or Hill number, reviewed by Lou Jost in 2006, 2008, 2010). Also, could consider a sensitivity analysis with Shannon’s (consider expressing as e^Shannon’s, which would be “effective number”). None of these are necessary. And I think you provide the data for interested readers to check themselves? Or it could be expanded on in subsequent publications by your group.

Thank you for the excellent suggestions on alternative ways to investigate discriminatory power. We did not include a range of diversity indices for simplicity, however the data are provided as part of the submission process as you noted and your suggestions are definite possibilities for future analyses.

Minor comments:
4) Abstract. “identification of CT genotypes is a necessity”. Consider “could be useful”. Trachoma may well be eliminated before chlamydia diversity is understood.

This is a valid point, the abstract has been changed (line 20).

5) Selecting samples with higher CT load might induce some bias, line 146-7. Could be mentioned in limitations.

This is a useful point and has been added to the limitations section, thank you (lines 682-685).
